# Perceptions of Animal Welfare on Livestock: Evidence from College Agronomy Students in Costa Rica

**DOI:** 10.3390/ani14101398

**Published:** 2024-05-07

**Authors:** Anthony Valverde, José Andrés González-Miranda, Francisco Sevilla, Sara Mora, Eduardo R. S. Roldan, Celso Vargas, Rodolfo González

**Affiliations:** 1 Laboratory of Animal Reproduction, Center for Research and Development in Sustainable Agriculture in the Humid Tropics, School of Agronomy, Costa Rica Institute of Technology (ITCR), San Carlos Campus, Alajuela 223-21002, Costa Rica; 2 Prosecutor’s Department, College of Psychologists of Costa Rica, San José 8238-1000, Costa Rica; prosocial.cr@gmail.com; 3 Doctorate in Natural Sciences for Development (DOCINADE), Costa Rica Institute of Technology (ITCR), San Carlos Campus, Alajuela 223-21002, Costa Rica; f.sevilla@itcr.ac.cr; 4 Faculty of Philosophy and Letters, School of Philosophy, National University (UNA), Omar Dengo Campus, Heredia 86-3000, Costa Rica; sara.mora.ugalde@una.ac.cr; 5 Department of Biodiversity and Evolutionary Biology, National Museum of Natural Sciences, Spanish National Research Council (CSIC), 28006 Madrid, Spain; roldane@mncn.csic.es; 6 School of Social Sciences, Costa Rica Institute of Technology (ITCR), Cartago Campus, Cartago 159-7050, Costa Rica; celvargas@itcr.ac.cr; 7 School of Languages and Social Sciences, Costa Rica Institute of Technology (ITCR), San Carlos Campus, Alajuela 223-21002, Costa Rica; crgonzalez@itcr.ac.cr

**Keywords:** animal sciences, undergraduate students, animal ethics, bio law, bioethics

## Abstract

**Simple Summary:**

This article focuses on students dedicated to the study of agronomy. To identify the general perception of students about animal welfare in production systems, four categories were developed and presented through a survey. The four constructs of animal welfare used in this study were as follows: (1) sociodemographic aspects; (2) generalities about animal welfare; (3) ethical and bioethical dimensions of animal welfare; and (4) animal welfare within the framework of bio law. The results indicate that most respondents agreed that animal welfare is important within animal production systems and that causing unnecessary suffering to animals is ethically questionable. Overall, the results indicated significant associations between sociodemographic factors and the different ethical perspectives related to animal welfare, concerning aspects of suffering and the use of animals in experimentation or entertainment activities. These sociodemographic factors mentioned included gender, educational level, and upbringing environment. Understanding the perceptions of university students in agronomy regarding animal welfare is the necessary substrate to improve on and update study programs related to animal production systems. This should be paramount in the training of future professionals who will manage animal welfare issues as part of their future careers in the livestock sector.

**Abstract:**

Ethical considerations regarding our treatment of animals have gained strength, leading to legislation and a societal focus across various disciplines. This is a subject of study within curricula related to agri-food sciences. The aim was to determine the perceptions of agronomy university students concerning animal welfare in livestock production systems. A survey was conducted to encompass various aspects, from participants’ sociodemographic attributes to their attitudes and behaviors regarding animal welfare and the consumption of animal products. Statistical analysis, performed using R software, delved into the associations between participants’ characteristics and their perspectives on the ethical, bioethical, and legal dimensions of animal welfare. Associations between demographic factors and ethical viewpoints among students were identified. Gender differences emerged in animal treatment perceptions, while rural and urban environments impacted perspectives on various animals. Bioethical considerations revealed distinctive disparities based on gender and education in concerns regarding animal welfare, value perceptions, evaluations of animal behaviors, and opinions on animal research. It is crucial to distinguish between animal welfare and the ethical considerations arising from coexisting with sentient beings capable of experiencing suffering. Ethical theories provide a lens through which we perceive our obligations toward animals. The responsibility to ensure animal welfare is firmly rooted in recognizing that animals, like humans, experience pain and physical suffering. Consequently, actions causing unjustified suffering or mistreatment, particularly for entertainment purposes, are considered morally unacceptable.

## 1. Introduction

Since the 1970s, the ethical concern regarding our relationship with animals has shifted from relatively limited [1] to progressively growing, almost exponentially, in recent times [2,3,4]. This surge has directly impacted legislation, making it increasingly protective of animal welfare [5]. Human ethics are crucial as they can justify obligations towards animals [6,7,8] and are rooted in Ethics or Moral Philosophy [9] by addressing imperatives that compel humans to act in specific ways [10]. Yet, understanding animal biology is necessary when delineating these obligations [11,12].

A significant ethical consideration is firmly rooted in society’s agreement that animals should not suffer [13,14]. However, the extent to which animals can be utilized as models in clinical trials for human pain treatment raises questions [15,16]. While not delving into animal suffering and consciousness, no harm should be caused to animals, including that which may arise from their participation in human entertainment activities [17,18,19]. Legal considerations, or alternatively, deontological aspects, further add to these debates [20]. The Five Domains Model for animal welfare assessment encompasses scientific issues related to this discipline [21]; however, it can also be used in conjunction with ethical theories to clarify our relationship with animals in various production or entertainment contexts [22,23,24].

It is also necessary to differentiate between animal welfare and the ethical issue arising from our coexistence with nonhuman sentient beings capable of experiencing physical suffering. Animal welfare that involves animals whose neuroendocrine systems are akin to those of humans should not exhibit physiological alterations. Emotional manifestations (e.g., fear or stress) should not deviate from what occurs under normal conditions [25,26,27]. Practically, welfare is assessed through the observation of animal behavior [28]. Positive human–animal relationships have been highlighted by several authors to contribute intrinsically to animal welfare [29,30,31,32].

The Five Domains Model (since 1994) for the assessment of animal welfare provides specific guidance on how to evaluate the negative and/or positive impacts of human behavior on animals [21]. This model facilitates the systematic and structured assessment of positive as well as negative welfare-related effects, the circumstances that give rise to them, and the potential interactions between both types of effects, all of which extend the utility of the model [33]. The Five Domains are clearly of use to animal behavior and welfare scientists because they can embrace new knowledge and understanding, as well as provide pointers for new studies. They can also be used for in-depth analysis of the impact of specific management practices (human actions) on animal welfare [34]. Industrial food animal production efficiently meets the demands for products globally, but often leads to animal welfare and health issues due to confinement in high densities. This is the point where ethical responsibility must arise, as well as a zootechnical commitment to ethical animal production, one that respects animals and upholds standards of productivity and quality [35,36,37].

Numerous ethical theories focus on the human–animal bond, with most centered around animal suffering [4,38]. The theory of reciprocal recognition suggests avoiding animal cruelty by recognizing the harm caused, considering both animal and human interests [39]. This ideology aligns with a derivative of utilitarianism [40,41], which advocates maximizing pleasure and minimizing suffering for sentient beings—forming the basis of ethics and obligations towards animals [18,42,43]. Such utilitarianism is common among professionals working with animals, aiming to prevent suffering and enhance animal welfare in livestock production [28,44].

The responsibility to ensure animal welfare is rooted in the established fact that animals experience physical pain and suffering [16,45]. Therefore, causing unjustified suffering, such as in entertainment involving cruelty, mistreatment, or unnatural conditions contrary to their physiological and behavioral needs, is not ethical and morally acceptable [6].

University education in agronomy promotes the human–animal bond in a practical academic learning environment, sensitizing students to animal welfare as they progress through their study programs [46], considering the sociodemographic attributes of the student population. This study aimed to determine the perceptions of agronomy university students concerning animal welfare in livestock production systems.

## 2. Materials and Methods

### 2.1. Data Source

The data used were collected from the Animal Welfare survey administered to students majoring in agronomy engineering at the Costa Rica Institute of Technology (ITCR) as part of an exploratory study on attitudes, behaviors, and perceptions toward animal welfare and meat consumption among agronomy engineering students at ITCR between May and July 2023. Informed consent was obtained from all the subjects involved in the study. The survey gathered a wide range of information, including the participants’ sociodemographic aspects, interaction with pet animals, general information about animal welfare, knowledge on the ethical dimension of animal welfare, consumption of animal products, consumption of meat and other animal-derived meat products, as well as attitudes toward meat products within animal welfare considerations. The data are available on the Open Science Framework. All interviewees were 18 years or older.

### 2.2. Data Collection

Data were collected in person through a survey. Prior to this, each participant was sent a consent form for participation, use of collected information, and data treatment within the framework of the research study. From the total respondents (N = 150), students who did not consent to participate in the research or were under 18 years old (n = 6) were excluded from the statistical analysis. A total of 144 surveys were analyzed.

The questionnaire comprised four parts distributed as follows: (1) Participants’ sociodemographic aspects, (2) Generalities on animal welfare, (3) Ethical and bioethics dimension of animal welfare, and (4) Animal welfare within the framework of bio-law.

To develop the questionnaire, recommendations were followed to maintain the wording as simple and direct as far as possible, using symmetric response scales, and framing elements both positively and negatively. Moreover, an expert consultation phase was included before the questionnaire’s administration. Regarding differences in response scale usage, the recommendations were to employ symmetric bipolar response scales with a clear midpoint.

### 2.3. Measures

In the present paper, the sociodemographic variables are gender, age, career level, upbringing environment, and income. The remaining questions were selected and categorized into three thematic groups as follows: (1) Ethical, consisting of 7 questions; (2) Bioethical, 5 questions, and (3) Legal, 5 questions. All responses are categorical.

The survey comprised questions related to the ethical, bioethical, and legal dimensions of animal welfare that were utilized in this study. Participants were asked various questions regarding animal use. Additionally, inquiries were made about suffering, sentience, and cruelty toward nonhuman animals, addressing the ethical, bioethical, and legal dimensions of animal welfare that extend beyond livestock production. Participants responded to items on a five-point Likert scale ranging from “1” (very good) to “5” (very bad). Subsequently, the responses were re-coded into “good” or “bad” and “yes” or “no” categories. 

The questions applied to the students were regarding their perception of animal ethics, bioethics, and legal issues.

Agronomy engineering students were surveyed on topics related to animal ethics to understand their perceptions based on sociodemographic variables such as gender, age, career level, geographic origin, and income. The questions are as follows:

- “In general, how do you believe farm animals are treated?”
- “Provide an assessment of how you believe the following farm animals are generally treated.”
- “Would you be willing to pay more for a product if it helped improve animal welfare?”
- “Do you believe humans should be respectful towards animals?”
- “Do you consider animal cruelty to be a condemnable behavior?”
- “Do you consider it ethical and responsible for social movements to aim at using ‘humane’ methods to exterminate crop pests?”
- “How do you assess the act of humans killing other animals for food?”

The questions related to life sciences and the interaction animal sciences and environment that could form the bioethical foundations of agronomy students were:

- “How would you rate your level of concern for animal welfare?”
- “Regarding animal suffering, do you believe some animals are more valuable than others?”
- “How do you assess nonhuman animals preying on each other?”
- “How do you assess animal research, specifically using them as models in trials to test new drugs for humans?”
- “How do you feel about the idea of witnessing an animal suffer?”

Questions related to the legal and regulatory aspects that could contribute to developing the argumentation on animal welfare within the framework of bio-law among agronomy students were as follows:

- “Do you find it ethical to practice “hunting” sports while complying with established regulations for it?”
- “Do you consider that animals subjected to hunting (even when each country’s regulations are followed) limit their well-being?”
- “If you admit that animals should not suffer, would you be willing to use animals in scientific experimentation as models for the study of human diseases?”
- “If you acknowledge that animals are not mere objects, do you believe that animals should be granted ‘rights’?”
- “Do you consider granting rights as the only way to protect the interests of animals?”

### 2.4. Statistical Analysis

Statistical analysis was performed in R version 4.3.1 [47] and RStudio version 2022.7.1.554 [48], using the packages ggplot2, version 3.5.1 [49] for figures and gtsummary, version 1.7.2 [50] for tables. The chi-squared test of independence was used to assess the association between the participants’ characteristics and each question within the thematic groups. In cases where the expected frequency was less than 5, the Fisher’s exact test was employed, and statistical significance was determined as a *p* value below 0.05. When skewness was present, the response categories were collapsed to the subsequent level.

### 2.5. Quantitative Analysis

Statistical analysis was conducted using R software (v4.2.2, R Core Team, 2021), and the summary statistics were computed for all variables of interest. Wilcoxon signed-rank tests were used to analyze all Likert scale questions to determine if the students’ responses differed among them. For each question, Wilcoxon signed-rank tests were conducted with the five Likert scale categories included (i.e., 1 = High; 2 = Medium or Low) or (1 = Yes, 2 = No, 3 = I am indifferent).

Based on the results of the Wilcoxon signed-rank test, only questions that were significantly different from each other (*p* < 0.05) were further analyzed. Ordinal logistic regression assuming proportional odds was employed to assess the relationship between Likert responses and the respondents’ demographic factors. Demographic factors considered for regression analysis were gender (male or female), age in years (18–20; 21–23; 24–26; over 26), progression level in the agronomy curriculum (less than 25%; between 25 and 50%; between 51 and 75%; over 75%), hometown type (rural or urban), agricultural background (originating from a farm or not), and experience with animals (yes or no). Model selection was performed through backward elimination based on parameter significance (*p* ≤ 0.05), retaining only significant parameters in the final models.

## 3. Results

### 3.1. Descriptive Statistics

**Figure 1:**
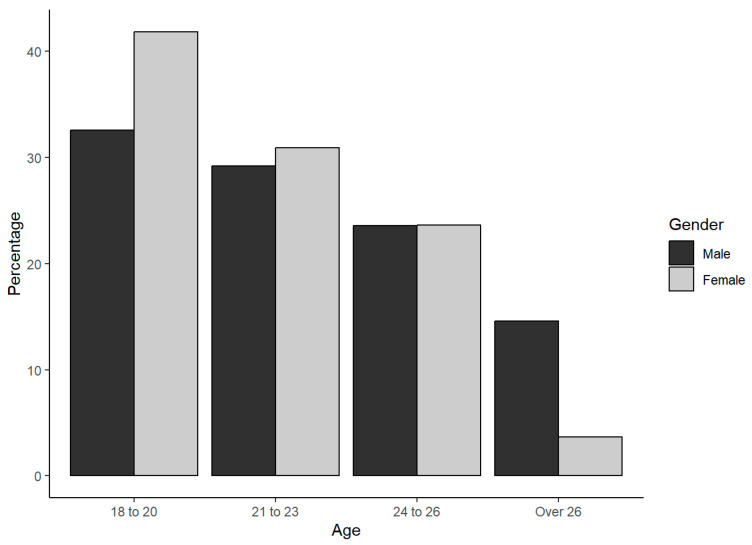
Sample age distribution for each gender.

**Figure 2:**
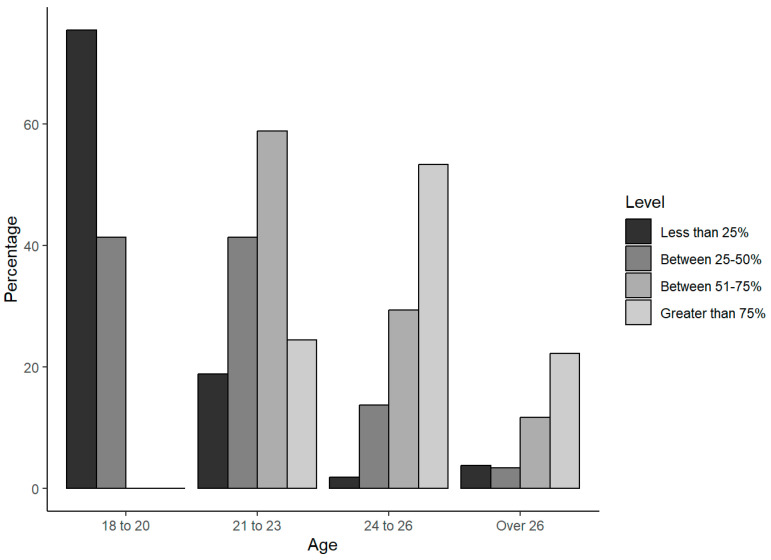
Age distribution within the college level.

A total of N = 150 individuals were contacted, of which only 144 met the inclusion criteria (above 18 years old) and were entered in the analysis, including n = 89 men and n = 55 women (Table 1). The age distribution for each gender is shown in the plot in Figure 1. As can be observed, the data are well balanced for gender, and there is a notable difference of around 10% within the 18 to 20 age group and a 38% difference among those over 26 years old, comprising 13 men and 2 women. The age distribution within the college level is shown in the plot in Figure 2. As expected of the career level, there is a clear trend—as the age increases, there is a higher percentage of individuals with advanced levels in their education.

### 3.2. Heat Maps Multivariate Data

#### 3.2.1. Perceived Animal Ethics

**Figure 3:**
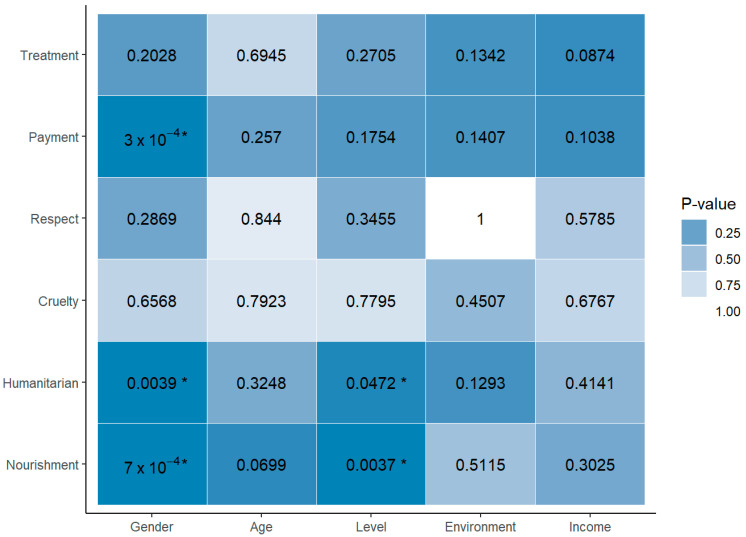
Heat map of the ethical perspectives and sociodemographic factors among agronomy engineering students. * *p* < 0.05.

The agronomy engineering students responded to questions regarding animal ethics aimed at understanding their perceptions based on sociodemographic variables such as gender, age, career level, upbringing environment, and income. The following heat map summarizes the association between the variables and questions, displaying the *p*-values of the hypothesis test. It can be concluded that gender and willingness to pay more for a product to improve animal welfare (“Would you be willing to pay more for a product if it helped improve animal welfare?”) are not independent. Similarly, gender and considering it ethical and responsible for social movements to use “humane” methods to exterminate pests (“Do you consider it ethical and responsible for social movements to aim at using ‘humane’ methods to exterminate crop pests?”), as well as the assessment of whether it is ethical for humans to kill animals for food (“How do you assess the act of human animals killing other nonhuman animals for food?”), are not independent. Likewise, the level of education and considering it ethical and responsible for social movements to use “humane” methods to exterminate crop pests, and the assessment of whether it is ethical for human animals to kill other nonhuman animals for food are not independent. In other relationships, there was not enough statistical evidence found to consider them non-independent (Figure 3).

The statistical associations between animal ethics perception and demographic factors (gender and upbringing environment) among the agronomy students about the question, “Provide an assessment of how you believe the following farm animals are generally treated”, are shown in Table 2. For turkeys, there is a statistically significant difference in perception between males and females (*p* = 0.016), indicating the varied viewpoints based on gender. The perception of beef cattle also significantly differs between genders (*p* = 0.050), showing distinct opinions among the male and female students.

When the upbringing environment was analyzed, perceptions of sheep significantly varied between the rural and urban areas (*p* = 0.050), suggesting differing viewpoints based on location. Likewise, the perceptions of rabbits, beef cattle, and dairy cattle also showcased notable differences between rural and urban settings. These findings indicate that both gender and upbringing environment influence the way agronomy students perceive various animals, with certain species’ perceptions being more significantly impacted by these demographic factors (Table 2). Other species were also included in the questionnaire, but the answers did not provide significant data, namely buffalo, goat, horse, sheep, rabbit, chicken, duck, and fish.

Concerning the question, “Would you be willing to pay more for a product if it helped improve animal welfare?” there exists, among the students, gender differences regarding the willingness to pay more for a product to improve animal welfare. The data demonstrate a substantial gender-based discrepancy (*p* < 0.001). Among men, 65% are inclined to consider paying more, with 49% expressing a definitive ‘Yes’ response. In contrast, women show a different trend, with 51% showing readiness to pay more, significantly higher than the 4.3% who oppose the idea. This gender-driven disparity highlights contrasting attitudes toward investing in animal welfare through higher product prices.

The ethical issues regarding the use of ‘humane’ methods in crop pest extermination across different demographics demonstrates a statistically significant difference (*p* = 0.004), where 82% of males oppose ‘humane’ pest control methods compared to the 18% of females who oppose it. Moreover, there is a variation (*p* < 0.001) across educational brackets. Participants within the final level of their career (>75%) exhibit less opposition (39%) compared to those within the early level of their career (<25%) at 25%. Regarding the perceptions of humans killing animals for food, there is a gender-based contrast (*p* < 0.001), where 71% of females view it negatively (‘Bad’) compared to 29% of males. There is a minor disparity (*p* = 0.070) across age groups. Younger participants (18–20) demonstrate higher negativity (62%) towards this act compared to older groups. Those students within the early level of their career (<25%) are more likely (57%) to perceive it negatively compared to those with higher education (>75%) at 4.8%. These findings indicate significant disparities in ethical considerations regarding crop pest control methods and perceptions of human animals killing nonhuman animals for food across different demographic segments (Table 3).

#### 3.2.2. Perceived Bioethics about Animal Welfare

**Figure 4:**
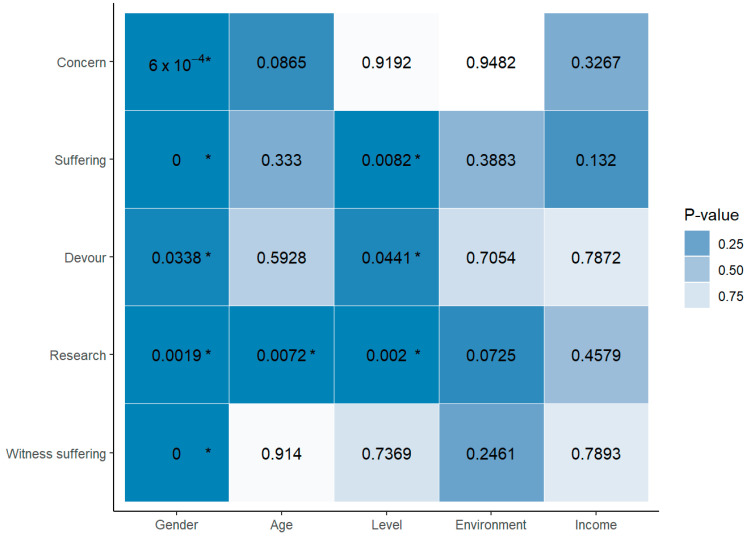
Heat map of the interdependencies between the sociodemographic variables and bioethical considerations in animal welfare by the agronomy students. * *p* < 0.05.

For the bioethics component, it may be concluded that each of the questions made in the bioethics component were not independent regarding gender. Similarly, there exists a relationship between age and the assessment of animal research, specifically between their use as models in trials for the testing of new drugs for humans (“How do you assess animal research, specifically using them as models in trials to test new drugs for humans?”) and the career level concerning the consideration that some animals are worth more than others (“Regarding animal suffering, do you believe some animals are more valuable than others?”); as well as between the assessment of nonhuman animals preying on each other (“How do you assess nonhuman animals preying on each other?”) and the evaluation of animal research, specifically their use as models in trials for testing new drugs for humans (“How do you assess animal research, specifically using them as models in trials to test new drugs for humans?”). In other relationships, there was insufficient statistical evidence to consider them as non-independent (Figure 4).

When the varying perspectives on animal welfare were analyzed, bioethical considerations, and the assessments regarding animal behaviors and research, highlighted gender and career level influences. Regarding animal welfare levels, there is a significant gender discrepancy (*p* < 0.001), with 83% of males rating their concern as “Low” compared to 47% of females. Similarly, the views on animal value perception reveal gender disparities (*p* < 0.001) and educational differences (*p* = 0.008). Furthermore, the assessments of animal behaviors show gender distinctions (*p* = 0.034) and educational variations (*p* = 0.044). Evaluating animal research as models for drug trials displays both gender (*p* = 0.002) and educational (*p* = 0.002) disparities. Additionally, differences between upbringing environment were noted, albeit marginally significant (*p* = 0.070) (Table 4).

When analyzing the responses related to the question, “How do you feel about the idea of witnessing an animal suffer?” all the indifferent respondents were men, while most females found it unbearable or unpleasant yet tolerable. This disparity reveals distinct gender-related perceptions regarding the witnessing of animal suffering, emphasizing a marked contrast in emotional reactions between male and female respondents (Table 5). No participant responded with “I enjoy it”.

#### 3.2.3. Perceived Legal and Normative Criteria Regarding Animal Welfare

**Figure 5:**
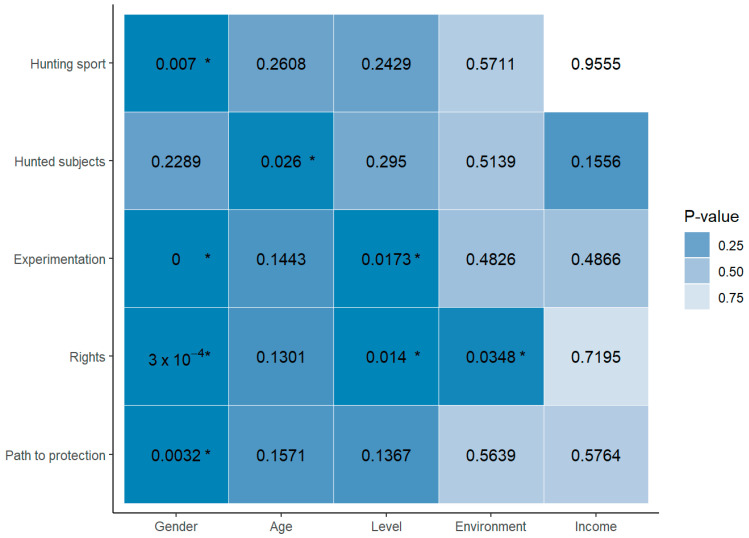
Heat map of the interrelated sociodemographic factors influencing the legal and normative criteria regarding the knowledge and perceptions on animal welfare of the agronomy students. * *p* < 0.05.

The relationships between gender and the bioethical perception of practicing “hunting” sports within established regulations (“Do you find it ethical to practice “hunting” sports while complying with established regulations for it? “), the willingness to use animals in scientific experimentation as models for the study of human diseases (“If you admit that animals should not suffer, would you be willing to use animals in scientific experimentation as models for the study of human diseases?”), considering whether animals should be granted “rights” (“If you acknowledge that animals are not mere objects, do you believe that animals should be granted ‘rights’?”), and believing that granting rights is the only way to protect animal interests (“Do you consider granting rights as the only way to protect the interests of animals?”) are not found to be independent (*p* < 0.05). Similarly, age and considering whether the hunting of animals (even when each country’s regulations are followed) limits their well-being (“Do you consider that animals subjected to hunting (even when each country’s regulations are followed) limit their well-being?”) are not independent (*p* < 0.05). Career level is also not independent (*p* < 0.05) of the willingness to use animals in scientific experimentation as models for the study of human diseases (“If you admit that animals should not suffer, would you be willing to use animals in scientific experimentation as models for the study of human diseases?”) and considering whether animals should be granted “rights” (“If you acknowledge that animals are not mere objects, do you believe that animals should be granted ‘rights’?”); likewise, a similar trend is observed regarding the upbringing environment with considering whether animals should be granted “rights” (“If you acknowledge that animals are not mere objects, do you believe that animals should be granted ‘rights’?”). In other relationships, there was insufficient statistical evidence to consider them non-independent (Figure 5).

Concerning the question about the ethical perception regarding practicing “hunting” sports in adherence to established regulations, the analysis highlights a statistically significant relationship (*p* < 0.05) between gender and responses (yes, no, indifferent to me), showing distinctions in the viewpoints between men and women. Specifically, 65% of the indifferent respondents were men, 52% of those who were against hunting to regulate overpopulation of species were male, and 82% of respondents in favor of such sports were also men.

When analyzing the activities regulated by legislation such as hunting to regulate overpopulation of species, recreational fishing, or crop pest control, differences were observed based on gender for each activity (*p* < 0.05). In general, there is a noticeable discrepancy between males and females in their assessments. For instance, in hunting to regulate overpopulation of species, a higher percentage of males rated it as “Good” compared to females. Similarly, in recreational fishing and pest control in crops, males tended to rate these activities higher compared to females (Table 6).

Regarding pest control in crops based on different career levels in college, there were significant differences observed among the groups (*p* < 0.001). The majority rated it positively across all categories. However, within the groups that assessed it as “Regular”, there was a notable discrepancy, with a higher percentage of respondents coming from the “51–75%” range completion of the curriculum in their agronomy degree compared to the other groups.

In examining perceptions about animal well-being in hunting scenarios, the effects (*p* < 0.05) of gender and age were revealed. Men tended to perceive hunting to regulate the overpopulation of a species as less limiting to animal well-being compared to women. Younger age groups were more likely to consider hunting to regulate the overpopulation of a species as affecting animal well-being. Regarding the use of animals in scientific research, there was an effect (*p* < 0.05) of both gender and career level exhibited. Men were more open about the idea of using animals in research compared to women, and those within the early level of their career were more inclined to support animal experimentation. Moreover, the effects of gender, career level, and upbringing environment on belief in granting animals ‘rights’ were displayed. Men were less likely than women to support granting animal’s rights, while being within the final level of their career and urban settings showed more favorable inclinations toward animal rights. The necessity of granting rights for animal protection also indicated gender disparities, with men being less inclined to view rights as the sole safeguard for animal interests compared to women (Table 7).

## 4. Discussion

Animal welfare is currently a fundamental component of animal production systems [3,38,51] because it directly impacts the quality of the meat, milk, eggs, wool, and leather industries and all their derivatives thereof. Typically, agronomical engineering schools in Costa Rica do not offer courses on animal welfare in their curricula; instead, they focus on livestock production in theoretical and practical courses on production systems. The results of this study indicate that most undergraduate agronomy students state that animal welfare is important within livestock production systems. This assessment aligns with the widespread social perception of how animals should be treated, where animal welfare involves reducing suffering in different livestock production systems [52]. The notion that causing animals to suffer is not acceptable is an ethical judgment that the science of animal welfare can utilize to promote the necessity for improving welfare to benefit the groups associated with the livestock industry and all recognized forms of human–animal relationships [53,54].

The results of this study highlight differences in animal welfare perceptions among different animal categories. The variability in students’ perceptions regarding the treatment of different animal species may be explained by the level of interaction individuals have with specific species due to their upbringing or environment. Various studies have demonstrated that experience in an agricultural or rural context and being male diminish perceptions of the importance of animal welfare [55,56,57]. However, several sociodemographic factors can influence individual perceptions of animal welfare. This underscores a somewhat variable sense of responsibility toward certain animal species, especially regarding wildlife or domestic species used in food production [58,59,60].

The aim of understanding the relevant perspectives which could underlie the perceptions and assessments of livestock is to propose new teaching activities [61], materials, and research [62]. We currently find two general positions with different variants as follows: the first one claims that human beings are different from the other species (we may call it anthropologism instead of anthropocentrism); there are some variants of this position [63,64]. Here, we adopt, without any further analysis, that there exists the biological species, as claimed by biological taxonomists for example [65].

The second position considers that human beings are part of the order of nature [66]. It is claimed that we share with other species most aspects including biological mechanisms, ingredients of life, and even cognitive structures [67]. It does not claim that the capabilities are the same along the natural order, but that different species share them with different degrees and differences [68].

When our results about perceptions were analyzed, most respondents held positive attitudes toward improving animal welfare [69,70]. These perceptions were linked to key concepts such as animal suffering, animal research, willingness to pay for products with guaranteed animal welfare, using ‘humane’ methods for crop pest control, and the moral conception of harvesting animals for meat consumption and animal-origin products. It is ethically desirable to address moral arbitrariness where they were not previously found, due to considering them as a natural variation [71]. If we acknowledge that animal suffering poses an ethical issue, we must investigate when and how animals suffer to address the problem based on evidence [14,16]. Here, we must employ the scientific method to conduct experiments associating the results with our current understanding in fields such as zoology, animal husbandry, genetics, evolutionary biology, and all sciences focused on living organisms.

Livestock production is often criticized for certain animal management practices, such as confinement systems, and procedures like castration or dehorning are considered controversial [72,73]. This awareness is evident among students and reflects in their ethical and bioethical perceptions regarding welfare and nonhuman animal suffering. However, this tends to increase as they progress through the agronomy curriculum. Regarding nonhuman animal suffering, it cannot be claimed that avoiding suffering makes a good person due to the recognition of their right “not to suffer”, because these are different categories [13] not elaborated in this article.

Attitudes toward animal welfare differed based on sociodemographic characteristics and the level of concern. Women, younger participants, and those students at an advanced level of the career showed higher levels of concern and held more negative attitudes toward animal abuse and suffering. They were also more likely to pay for animal-origin products with welfare seals (WTP—willing-to-pay) and welfare-friendly products (WFP), indicating the awareness of responsibly consuming animal-origin products and a potential market consciousness of the value animals provide as protein sources in human diets [44,74]. Women held more negative attitudes regarding animal research involving their suffering and expressed greater concerns for animal welfare. Regarding gender, some authors highlight the role of women, the feminist ethics [75], and their solidarity-driven roles in society [76], which could explain their more empathetic perceptions about welfare concerns compared to men. The students entering agronomy programs might have increased access to information about production systems through social media, influencing their level of concern for animal treatment. However, this study demonstrates that the perceptions of agricultural production systems tend to change more positively as the students’ progress through the curriculum. This change could be associated with advanced-level courses on animal production, sensitising their perception objectively towards the treatment of animals in different livestock species, as well as caring more for the wellbeing of the species they will continue to interact with in livestock production. Further supporting the role of social media in agricultural education, a notable example is a study where Instagram was used effectively to educate and engage the public about dairy cow nutrition and welfare. This initiative successfully communicated scientific knowledge to a wider audience, creating a community engaged in the welfare of dairy cows. Such innovative approaches in digital outreach highlight the evolving landscape of agricultural education and the growing importance of accessible and interactive platforms for public engagement [77].

Analyzing the perceptions of animal welfare based on sociodemographic variables could be explained through the human–animal bond. An interdisciplinary approach to addressing this bond could generate new knowledge related to the bioethics of animal welfare, where ethical issues about our relationship with animals [6,10] interact with the knowledge of animal biology [78], the latter being something that agronomy students learn in the early years of their program. The bioethical principle of autonomy recognizes the telos, i.e., the fundamental biological and psychological essence of any animal based on Aristotle’s principles [9]. According to this doctrine, the purposes of humans and nonhuman animals do not need coincide as the decision to ethically treat animals is a moral stance specific to humans, therefore, it is not a biological purpose but an ethical decision [79]. In accordance with the ethics of animal husbandry, nonhuman animals currently serve rational human purposes through breeding and good livestock practices.

Sociodemographic variables such as gender, students’ upbringing environment, and their progress within the curriculum directly influenced ethical and bioethical perceptions regarding the human–animal bond and animal welfare. In this study, most students (85%) came from rural backgrounds compared to 15% from urban areas. This differs from data reported by some authors that underscore a current trend regarding the backgrounds of students studying agronomical sciences who are not from rural environments [80,81]. The correlation between sociodemographic variables and the degree of ethical concern for animal welfare might stem from the animal interest movement that gained momentum after the publication of the book ‘*Animal Liberation*’ [82], marking a turning point in civil society who grow increasingly concerned about how animals are treated [83,84,85,86] on farms or livestock production facilities [23,87].

The equal consideration of interests introduced by Peter Singer in 1975 (Animal Liberation) is an interesting qualification of nonhuman animals. He introduces it as an ethical principle and not as a description of facts. What this does is prescribe the correct way of understanding and of behavior that we should adopt in connection with other living organisms [88].

The educational level was linked to greater concern for welfare, with those who had studied longer expressing more affinity and concern for it. Those with more technical knowledge also reported higher concerns, similar to those more familiar with the concept of animal welfare, for instance, those who previously lived in rural areas, owned farms, or worked in livestock activities. This explains how prior knowledge of activities can sensitize attitudes and perceptions, along with knowledge transmitted through formal education, to avoid misconceptions about animal production that could potentially affect attitudes.

Some studies have analyzed the sociodemographic characteristics and their relation to perceptions and attitudes about animal welfare, and none have reported significant differences attributable to socioeconomic factors, such as in the present study with family income level [89,90]. Students’ preferences indicate that they would pay a different price to improve welfare. Although the effect was significant when analyzing gender, this could provide market niches and should be analyzed in association with producers to assess the costs associated with animal welfare and the benefits of investments made following the ethical criteria. Some studies suggest the need for interdisciplinary work because it is challenging to generate a global concept of welfare that satisfies all involved parties [91]. The best way to build a just multispecies society may not be known in the present moment, but accepting this responsibility will positively impact the journey to find it out [92].

Negative perceptions and concerns associated with animal welfare were confined to a zoocentric or anthropocentric view. Perceptions about welfare from an animal perspective are based on the animals’ ability to express emotions and feel pain [10], ergo, the observable behavior that would elicit an uncomfortable response in humans. In this regard, the perception that animals may have a value greater than the utilitarian value for humans becomes relevant. However, the utilitarian argument for animal welfare lies in the fact that subsequent animal products will be better for quality and the safety of the humans consuming them. Conversely, some people’s concerns about animal welfare were motivated by their own well-being, seemingly avoiding anthropomorphism. This was reflected in the educational levels; in the initial years of the program, there was a negative perception of meat consumption, and as students progressed in their university careers, this perception significantly shifted positively toward meat consumption. Understanding livestock production systems might sensitize this perception change. However, the criterion that animals are treated inadequately or even cruelly remained constant. Some authors have described consuming animal meat as a human right [93]. The centric view can also offer another form of dissonance, seeing animals as objects rather than sentient beings [64,94]. This constitutes a scientific problem and should be addressed as such, without our opinions on animal rights or obligations intervening or interacting. Welfare legislation should consider these scientific issues and this will likely affect production systems because consumers are concerned about the welfare of farm animals, leading to social pressure that will affect new legislation. Certain production systems are also being phased out, such as that of foie gras, where force-feeding induces fatty liver in ducks and geese to obtain the “delicacy” product, an act that could be labeled as barbaric [95].

Although most students did not have negative perceptions of the regulated activities like hunting to control species overpopulation, recreational fishing, and pest control, this could be explained by their interactions within agronomy programs involving courses related to agricultural crops, where they engage in supervised practices for biological and/or chemical pest control in crops [96,97]. Animals related to fishing and hunting to regulate the overpopulation of a species, not livestock species, might denote a gradient in the value attributed to species closer to humans due to the human–animal bond [13], for example, mammals. The scales for assessing suffering established a gradient with primates at the forefront, followed by pets, and then livestock animals.

Concerning animal rights, experimentation with them, and the ways to protect animal interests, these considerations were not independent of gender, educational level, or students’ upbringing environments. Students’ assessments regarding bio-right-related questions suggest a positive stance regarding granting rights to animals, with 70% of respondents indicating that rights should be granted. Granting rights to animals is a complex issue studied in various works [64,92,98,99,100]. However, granting rights should go hand in hand with acquiring duties [101,102], and the predominant question is: Can rights be granted without assuming duties? From a broader perspective, production animals exist under the biopower exerted upon them by the human species, an act defined by Foucault [103,104], such as the subjugation and disposal of their bodies and population control. Therefore, discussions about animal welfare should be directed toward recognizing this power relationship and seeking to reduce the mistreatment linked to domination, rather than aiming for a ‘liberation’ from the breeding and production process for harvesting and generating animal-origin foods. Biopower also functions as a technology of the humanist ideological system to differentiate people from the rest of the animals, as our rationality both dominates and excludes them from the social contract. This implies that animals are not accountable for their behaviors within a human ethics system that monitors behaviors and punishes them, yet they also do not possess the rights or sovereignty over their bodies [105]. It is incongruous to grant rights to production animals because they exist to be ‘utilized’ by the human species, and they could never emancipate themselves from this power relationship. Even attempting to grant them rights is another act of domination through rationality and social agreement systems. Instead, it is important for the dominant species to ensure their welfare from the inherent responsibility and the moral duty to prevent harm and, furthermore, from a zootechnical commitment to responsible animal production that respects animals and ensures productive quality.

## 5. Conclusions

Animal welfare is a topic of increasing relevance in society, as there is growing concern about responsible consumption habits regarding animal-origin products. This concern even impacts legislation regarding nonhuman animal use. Consequently, public perceptions about animal welfare are complex and often understudied within various professional groups associated with animals. Overall, the findings of this study suggest that most respondents hold positive ethical assessments toward animal welfare and negative perceptions toward animal suffering. However, differences emerged when consulting about pest control among species. It has been demonstrated that perceptions of animal welfare vary among species, genders, levels of university education, and places of origin. This highlights the importance of understanding agronomy students’ perceptions of animal welfare as the input for designing, improving, and updating educational programs with formal components on animal welfare. An integrated education on animal welfare could equip students with multidisciplinary knowledge supporting the science of animal welfare to address the current challenges and dilemmas concerning animal welfare.

## Figures and Tables

**Table 1:** Number of respondents (percentage variation in brackets) to the questions about animal ethics, bioethics, and legal issues in animal welfare according to sociodemographic variables (N = 144).

Gender		Age Breakdown				College Level		Upbringing Environment	
Male	Female	18–20	21–23	24–26	>26	<50%	>50%	Rural	Urban
89 (62)	55 (38)	52 (36)	43 (30)	34 (24)	15 (10)	82 (57)	62 (43)	123 (85)	21 (15)

**Table 2:** Ethical perception on animal welfare among agronomy students regarding the question “Provide an assessment of how you believe the following farm animals are generally treated”.

Animal			*p*-Value ^2^
	Gender		
	Male, n = 89 ^1^	Female, n = 55 ^1^	
Turkey			0.016
Bad	10 (11%)	0 (0%)	
Regular	36 (40%)	29 (53%)	
Good	43 (48%)	26 (47%)	
Beef cattle			0.050
Bad	4 (4.5%)	9 (16%)	
Regular	35 (39%)	21 (38%)	
Good	50 (56%)	25 (45%)	
Dairy cattle			0.10
Bad	6 (6.7%)	9 (16%)	
Regular	23 (26%)	17 (31%)	
Good	60 (67%)	29 (53%)	
	Upbringing environment		
	Rural, N = 123 ^1^	Urban, N = 21 ^1^	
Sheep			0.050
Bad	3 (2.4%)	2 (9.5%)	
Regular	51 (41%)	12 (57%)	
Good	69 (56%)	7 (33%)	
Rabbit			0.026
Bad	6 (4.9%)	4 (19%)	
Regular	34 (28%)	8 (38%)	
Good	83 (67%)	9 (43%)	
Beef cattle			0.039
Bad	8 (6.5%)	5 (24%)	
Regular	48 (39%)	8 (38%)	
Good	67 (54%)	8 (38%)	
Dairy cattle			0.050
Bad	10 (8.1%)	5 (24%)	
Regular	33 (27%)	7 (33%)	
Good	80 (65%)	9 (43%)	

^1^ n (%), ^2^ Fisher’s exact test; Pearson’s chi-squared test.

**Table 3:** Ethical perspectives on ‘humane’ pest control and human–animal interactions surrounding food practices—gender and career level influences.

“Do You Consider It Ethical and Responsible for Social Movements to Aim at Using ‘Humane’ Methods to Exterminate Crop Pests?”			
Variable	No, n = 44 ^1^	Yes, n = 35 ^1^	*p*-Value ^2^
Gender			0.004
Male	36 (82%)	18 (51%)	
Female	8 (18%)	17 (49%)	
Career level			
25%	11 (25%)	17 (49%)	
25–50%	10 (23%)	10 (29%)	
51–75%	6 (14%)	1 (2.9%)	
>75%	17 (39%)	7 (20%)	
“How do you assess the act of humans killing other animals for food?”			
Variable	Bad, n = 21 ^1^	Good, n = 123 ^1^	*p*-value ^2^
Gender			<0.001
Male	6 (29%)	83 (67%)	
Female	15 (71%)	40 (33%)	
Age			0.070
18–20	13 (62%)	39 (32%)	
21–23	5 (24%)	38 (31%)	
24–26	2 (9.5%)	32 (26%)	
>26	1 (4.8%)	14 (11%)	
Career level			0.004
<25%	12 (57%)	41 (33%)	
25–50%	3 (14%)	26 (21%)	
51–75%	5 (24%)	12 (9.8%)	
>75%	1 (4.8%)	44 (36%)	

^1^ n (%), ^2^ Fisher’s exact test; Pearson’s chi-squared test. Percentages within each variable are shown for each column.

**Table 4:** Influential factors in attitudes toward animal welfare, bioethical perceptions, and assessments in agronomy students.

How Would You Rate Your Level of Concern for Animal Welfare?			
Variable	Low, n = 42 ^1^	High, n = 102 ^1^	*p*-Value
Gender			<0.001
Male	35 (83%)	54 (53%)	
Female	7 (17%)	48 (47%)	
Regarding animal suffering, do you believe some animals are more valuable than others?			
Variable	No, N = 63 ^1^	Yes, N = 81 ^1^	*p*-value ^2^
Gender			<0.001
Male	26 (41%)	63 (78%)	
Female	37 (59%)	18 (22%)	
Career level			0.008
<25%	32 (51%)	21 (26%)	
25–50%	11 (17%)	18 (22%)	
51–75%	8 (13%)	9 (11%)	
>75%	12 (19%)	33 (41%)	
How do you assess nonhuman animals preying on each other?			
Gender			*p*-value ^2^
Variable	Bad, n = 16 ^1^	Good, n = 128 ^1^	0.034
Male	6 (38%)	83 (65%)	
Female	10 (63%)	45 (35%)	
Career level			0.044
<25%	7 (44%)	46 (36%)	
25–50%	2 (13%)	27 (21%)	
51–75%	5 (31%)	12 (9.4%)	
>75%	2 (13%)	43 (34%)	
How do you assess animal research, specifically using them as models in trials to test new drugs for humans?			
Variable	Bad, n = 84 ^1^	Good, n = 60 ^1^	*p*-value ^2^
Gender			0.002
Male	43 (51%)	46 (77%)	
Female	41 (49%)	14 (23%)	
Career level			0.002
<25%	37 (44%)	16 (27%)	
25–50%	22 (26%)	7 (12%)	
51–75%	7 (8.3%)	10 (17%)	
>75%	18 (21%)	27 (45%)	
Upbringing environment			0.070
Rural	68 (81%)	55 (92%)	
Urban	16 (19%)	5 (8.3%)	

^1^ n (%), ^2^ Fisher’s exact test; Pearson’s chi-squared test.

**Table 5:** Gendered perceptions and emotional disparities among the agronomy students about animal suffering.

Variable	Indifferent n = 8 ^1^	Unbearable n = 68 ^1^	Unpleasant Yet Tolerable n = 68 ^1^	*p*-Value ^2^
Gender				<0.001
Male	8 (100%)	29 (43%)	52 (76%)	
Female	0 (0%)	39 (57%)	16 (24%)	

^1^ n (%), ^2^ Fisher’s exact test; Pearson’s chi-squared test.

**Table 6:** Perceptions of gender-associated regulated activities: hunting to regulate overpopulation of species, recreational fishing, and pest control in crops by agronomy engineering students.

Activity			*p*-Value ^2^
	Gender		
	Male, n = 89 ^1^	Female, n = 55 ^1^	
Hunting to regulate overpopulation of species			0.005
Bad	16 (18%)	19 (35%)	
Regular	21 (24%)	19 (35%)	
Good	52 (58%)	17 (31%)	
Recreational fishing			<0.001
Bad	7 (7.9%)	17 (31%)	
Regular	29 (33%)	24 (44%)	
Good	53 (60%)	14 (25%)	
Pest control in crops			<0.001
Bad	1 (1.1%)	1 (1.8%)	
Regular	8 (9.0%)	18 (33%)	
Good	80 (90%)	36 (65%)	

^1^ n (%), ^2^ Fisher’s exact test; Pearson’s chi-squared test.

**Table 7:** Gender disparities in the perceptions of animal well-being associated with insights from hunting to regulate the overpopulation of species, research, and animal rights advocacy.

“Do You Consider that Animals Subjected to Hunting (Even When Each Country’s Regulations Are Followed) Limit Their Well-Being?”			
Variable	No, n = 19 ^1^	Yes, n = 80 ^1^	*p*-Value ^2^
Gender			0.2
Male	14 (74%)	47 (59%)	
Female	5 (26%)	33 (41%)	
Age (years)			0.026
18–20	4 (21%)	31 (39%)	
21–23	4 (21%)	28 (35%)	
24–26	10 (53%)	14 (18%)	
>26	1 (5.3%)	7 (8.8%)	
“If you admit that animals should not suffer, would you be willing to use animals in scientific experimentation as models for the study of human diseases?”			
Variable	No, N = 86 ^1^	Yes, N = 58 ^1^	*p*-value ^2^
Gender			<0.001
Male	41 (48%)	48 (83%)	
Female	45 (52%)	10 (17%)	
Career level			0.017
<25%	39 (45%)	14 (24%)	
25–50%	17 (20%)	12 (21%)	
51–75%	11 (13%)	6 (10%)	
>75%	19 (22%)	26 (45%)	
“If you acknowledge that animals are not mere objects, do you believe that animals should be granted ‘rights’?”			
Variable	No, N = 43 ^1^	Yes, N = 100 ^1^	*p*-value ^2^
Gender			<0.001
Male	36 (84%)	52 (52%)	
Female	7 (16%)	48 (48%)	
Career level			0.014
<25%	11 (26%)	42 (42%)	
25–50%	5 (12%)	24 (24%)	
51–75%	6 (14%)	10 (10%)	
>75%	21 (49%)	24 (24%)	
Upbringing environment			0.035
Rural	41 (95%)	82 (82%)	
Urban	2 (4.7%)	18 (18%)	
“Do you consider granting rights as the only way to protect the interests of animals?”			
Variable	No, N = 97 ^1^	Yes, N = 47 ^1^	*p*-value ^2^
Gender			0.003
Male	68 (70%)	21 (45%)	
Female	29 (30%)	26 (55%)	

^1^ n (%), ^2^ Fisher’s exact test; Pearson’s chi-squared test.

## Data Availability

The data presented in this study are available within the article.
